# Combined Effect of HF‐rTMS and Whole‐Body Vibration Exercise on Cognitive Efficiency in Esports Players With or Without Sedentary Behaviors: A Randomized Controlled Trial

**DOI:** 10.1002/brb3.70473

**Published:** 2025-05-08

**Authors:** Shan He, Lu Leng, Dongdong Gao, Yu Chen, Weiji Deng, Jiarui Wu, Peilun Li, Yilin Chen, Jinglin Huang, Guoqing Liu, Jiarui Su, Jianwei Peng, Wenhuang Guo, Junfeng Zhang, Junhao Huang

**Affiliations:** ^1^ Guangdong Provincial Key Laboratory of Physical Activity and Health Promotion, Scientific Research Center Guangzhou Sport University Guangzhou Guangdong China; ^2^ College of Foreign Languages Jinan University Guangzhou Guangdong China; ^3^ Dr. Neher's Biophysics Laboratory for Innovative Drug Discovery, State Key Laboratory of Quality Research in Chinese Medicine Macau University of Science and Technology Macau China

**Keywords:** cognitive performance, electronic sports, sedentary behavior, transcranial magnetic stimulation, whole‐body vibration training

## Abstract

**Background:**

The present study investigated the effects of high‐frequency repetitive transcranial magnetic stimulation (HF‐rTMS), whole‐body vibration training (WBVT), and a combination of HF‐rTMS and WBVT interventions on cognitive performance in esports players with or without sedentary behaviors.

**Methods:**

A total of 128 participants, including sedentary and non‐sedentary esports players, were randomly assigned to the HF‐rTMS group, the WBVT group, the HF‐rTMS + WBVT group, or the control group. The interventions were administered daily for 2 weeks, and pretest, mid‐test, and posttest assessments were conducted. Cognitive function was assessed using the Digit Symbol Substitution Test (DSST) for response time and accuracy, and gaming performance was measured using first‐person shooter (FPS) scores.

**Results:**

At baseline, sedentary players demonstrated significantly increased response time in DSST compared to non‐sedentary participants. The interventions, both HF‐rTMS and WBVT, significantly enhanced cognitive processing speed and accuracy, with these improvements being more pronounced in sedentary esports players compared to non‐sedentary esports players. Notably, the combination of HF‐rTMS and WBVT was found to be the most effective in boosting cognitive performance among these interventions. Furthermore, FPS scores showed an overall increase in all intervention groups in both sedentary and non‐sedentary esports players, and the combination of HF‐rTMS and WBVT showed the most pronounced effect on in gaming performance.

**Conclusion:**

The study demonstrated that sedentary behavior had a detrimental effect on the cognitive function in esports players. Furthermore, HF‐rTMS and WBVT, especially in combination, effectively enhanced cognitive performance in esports players, with a more pronounced effect in those with sedentary lifestyles. These findings suggested potential strategies for cognitive enhancement in the esports context, highlighting the need for tailored interventions based on individual lifestyle factors.

## Graphical abstract

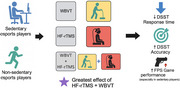

## 1 Introduction

Since its emergence in the 1990s, electronic sports (esports), which refers to organized competitive gaming, have grown exponentially, both in popularity and in global reach. As of 2023, the number of global esports players is projected to reach 3.38 billion, contributing to an estimated industry revenue of 184.0 billion U.S. dollars (Newzoo, n.d.). This rapid expansion has transformed esports into a major global industry, attracting players, enthusiasts, and sponsors from all corners of the world (Freeman and Wohn 2017; Southern 2017). With the increasing competitiveness of the field, there is a growing demand for skilled players who not only excel in gameplay but also demonstrate superior cognitive abilities.

Cognitive functions, such as executive functioning, working memory, attention, and processing speed, are integral to performance in esports (Hagiwara et al. 2020; Pedraza‐Ramirez et al. 2020). Quick decision‐making, efficient strategy execution, and precise motor control are essential components of esports gameplay, all of which heavily rely on cognitive skills (Valls‐Serrano et al. 2022). Esports requires not only cognitive skills such as memory, attention, and executive function but also physical skills, such as hand‐eye coordination, reaction time (RT), and spatial awareness (Sousa et al. 2020; Beres et al. 2023). As such, interventions that enhance both cognitive and physical abilities may have the most profound effects on gaming performance. Therefore, understanding and enhancing cognitive performance in esports players has become a significant area of interest for researchers, coaches, and players alike, as these abilities directly impact competitive success. Supporting Information.

In recent years, however, concerns have emerged regarding the sedentary behavior prevalent among esports players. Extended hours of practice and competition in front of screens have led to a lifestyle characterized by prolonged sitting and limited physical activity (Toth et al. 2020). This sedentary behavior poses potential risks to both physical health and cognitive performance (Chang et al. 2012; Choi et al. 2021), with research indicating that it may impair critical cognitive functions, such as attention, processing speed, and working memory (Lee et al. 2020; Sainz et al. 2020). As esports players increasingly face the dual challenge of maintaining both cognitive performance and physical health, addressing the consequences of sedentary behavior has become an important focus for improving overall performance in the field.

Over the past few years, several studies have explored various cognitive enhancement techniques to improve performance in competitive gaming. Research has shown that cognitive training, such as working memory and attention tasks, can lead to improvements in gaming performance (Green and Bavelier 2012; Moisala et al. 2017). A study examined the application of noninvasive brain stimulation (NIBS) in esports, highlighting its potential to enhance motor and cognitive skills essential for gaming (Zhuang et al. 2020). Similarly, Campbell and Toth (2021) investigated the effects of expertise, training, and neurostimulation on sensory‐motor skills in esports. Additionally, techniques like neurofeedback and transcranial direct current stimulation (tDCS) have been investigated for their potential to modulate brain activity and improve cognitive function in gaming contexts (Toth et al. 2020; Yan et al. 2020). However, although these approaches have demonstrated promising results in enhancing cognitive abilities such as attention and memory, there is limited evidence regarding their long‐term effects and overall efficacy in esports (Hoy and Fitzgerald 2010).

High‐frequency repetitive transcranial magnetic stimulation (HF‐rTMS) has gained significant attention for its capacity to modulate neural activity. This noninvasive technique leverages magnetic fields to activate distinct cortical regions of the brain (Hallett 2007). Although its initial exploration centered on treating a spectrum of neurological and psychiatric conditions, for example, depression (George and Aston‐Jones 2010), its potential to bolster cognitive capacities, spanning attention, memory, and executive functions, has garnered increasing recognition (Guse et al. 2010). Meanwhile, whole‐body vibration training (WBVT), traditionally well‐known for its musculoskeletal advantages, has recently been associated with cognitive enhancements (Rittweger 2010). During WBVT, participants stand on oscillating platforms, inducing rapid muscular contractions and potentially driving neuromuscular adaptability. Beyond its evident physical perks, elevated levels of brain‐derived neurotrophic factor (BDNF) following WBVT hint at potential neural benefits, which could influence cognitive dynamics (Ferris et al. 2007). Unlike other cognitive enhancement techniques, the combined approach of HF‐rTMS and WBVT offers a more holistic and potentially synergistic method for enhancing cognitive and physical performance in esports players.

Recent investigations into the applications of HF‐rTMS and WBVT reveal notable findings. HF‐rTMS, particularly when targeting the dorsolateral prefrontal cortex (DLFPC), demonstrates enhanced motor coordination in athletes, as evidenced in a study involving female volleyball players (Moscatelli et al. 2023). Concurrently, WBVT emerges as a promising intervention in athletic conditioning, with reviews suggesting its efficacy in improving performance (Costantino et al. 2014). Intriguingly, the benefits of WBVT extend to sedentary populations, particularly overweight/obese women, where it significantly ameliorates arterial stiffness and muscle strength (Alvarez‐Alvarado et al. 2017). Although direct research on the combined use of HF‐rTMS and WBVT is scant, a study combining HF‐rTMS with cognitive training in a cerebral ischemic rat model hints at potential cognitive improvements, warranting further exploration into this synergistic approach (Hong et al. 2021). These findings underscore the multifaceted benefits of these modalities, meriting continued research, particularly in their combined application for cognitive enhancement.

Neuroplasticity theories (Brown 1998) provide a biological explanation for the effects of both interventions. Neuroplasticity refers to the brain's ability to reorganize itself by forming new neural connections in response to stimuli. HF‐rTMS has been shown to enhance neuroplasticity by increasing the excitability of cortical neurons, particularly in areas related to motor coordination and executive function. WBVT, on the other hand, has been linked to increases in BDNF, which is known to support the growth and survival of neurons. These interventions, therefore, may improve the brain's capacity for complex cognitive tasks, directly benefiting esports performance, where fine motor control and rapid cognitive processing are critical.

In the context of esports, where milliseconds matter, improved cognitive functions from HF‐rTMS and WBVT could be pivotal. The capacity to think faster, react swiftly, and endure longer gaming sessions without fatigue can be significant determinants of esports player's success (Pedraza‐Ramirez et al. 2020). However, application of these interventions on esports professionals is an area that remains largely uncharted (Dalhuisen et al. 2023).

Although HF‐rTMS has proven effective in clinical settings for neurological disorders, its application in sports and gaming remains controversial. Some argue that such interventions may provide an unfair advantage by artificially enhancing cognitive performance, raising ethical concerns about the potential use of these methods as a form of “doping” in competitive gaming (Perez‐Trivino 2018). Additionally, questions about the optimal duration and intensity of WBVT sessions, as well as its potential for causing physical strain or discomfort, warrant further investigation (Minhaj T et al. 2022).

A stronger link between sedentary behavior and esports performance is needed to justify applying interventions like HF‐rTMS and WBVT. Although general cognitive abilities are important, the specific cognitive measures targeted by these interventions have been chosen due to their relevance in enhancing both motor coordination and mental endurance, two factors that are critical in esports. Furthermore, research on these modalities has demonstrated their effectiveness in improving performance and overall health in both active and sedentary populations, which strengthens the case for their application in esports. Given the sedentary nature of many esports players, these interventions could be particularly beneficial in optimizing cognitive function and mitigating the negative effects of prolonged inactivity. The present study aims to explore how HF‐rTMS and WBVT can enhance cognitive performance in esports players, particularly those with sedentary behaviors. By enhancing our understanding of these interventions’ effects on cognitive performance and exploring their application in the esports domain, the present study has the potential to contribute valuable insights for the development of tailored interventions and training programs to optimize cognitive abilities in esports players with or without sedentary behaviors.

## 2 Methods

### 2.1 Study Design and Participants

**FIGURE 1:**
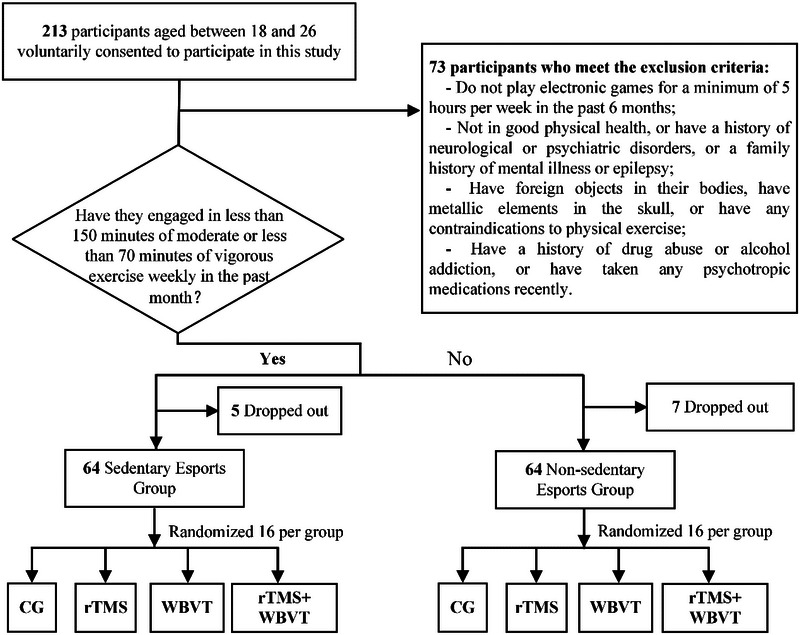
The flowchart of the program.

A total of 213 voluntary participants, all university students, aged between 18 and 26, were initially included in this study. However, 140 individuals met the inclusion criteria: having played electronic games for a minimum of 5 h per week over the last 6 months, being in good physical health without a history of neurological or psychiatric disorders or a family history of mental illness or epilepsy, having no foreign objects in their bodies or metallic elements in the skull, and having no contraindications to physical exercise, and without a history of drug abuse, alcohol addiction, or recent psychotropic medication use (Figure 1).

The eligible 140 participants were then categorized into two groups based on their level of physical activity in the past month. Participants who had engaged in less than 150 min of moderate‐intensity exercise or 70 min of vigorous‐intensity exercise per week were categorized as the sedentary esports group. The remaining participants formed the non‐sedentary esports group. However, five individuals from the sedentary esports group and seven individuals from the non‐sedentary esports group dropped out of the study due to various physical reasons and other issues. Finally, a total of 128 participants, including sedentary and non‐sedentary esports players, were randomly assigned to the HF‐rTMS group, the WBVT group, the HF‐rTMS + WBVT group, or the control group.

### 2.2 Experimental Intervention

In a meticulously controlled environment, the study was conducted in professional psychological examination suites with complementary laboratory settings, ensuring minimal external perturbations. Upon arrival, participants received comprehensive details regarding the study, inclusive of the physical activity scale. The investigator elucidated both the gaming and testing task demands and experimental protocols prior to initiation. A preliminary practice session lasting approximately 10 min ensured participants’ acclimation to the study rules before the formal commencement.

Each intervention was applied daily, maintaining consistent timing over a 2‐week period. Participants engaged in WBVT on a specialized vibrating apparatus, specifically the Power Plate my5, utilizing My5R Vibration Technology. Positioning involved standing with a slight foot separation, and handrails ensured stability. The protocol encapsulated six 2‐min passive WBVT sessions at 30 Hz with an amplitude of approximately 0.5 mm, interspersed with six 2‐min nonvibratory control sessions.

Participants underwent HF‐rTMS by employing the M‐100 Ultimate Pulsed Magnetic Stimulation Device (Shenzhen Yingchi Technology Co. Ltd., Shenzhen, China). Preliminary assessments determined each participant's resting motor threshold (RMT) via single‐pulse TMS. RMT is demarcated as the minimal stimulus magnitude eliciting motor‐evoked potential (MEP) amplitudes ≥50μV in a minimum of 5 out of 10 trials at muscular rest. The HF‐rTMS target was the left DLFPC (denoted as F3 in the 10–20 system), owing to its documented functional lateralization in previous reports (Curtin et al. 2019). For the HF‐rTMS procedure, participants were comfortably seated, utilizing earplugs, and instructed to remain composed and vigilant. Their forearm positioning was natural, lightly resting on their thighs with upward‐facing palms and relaxed fingers. Throughout the HF‐rTMS, meticulous attention was paid to maintain the coil's orientation and positioning, ensuring it remained within the acceptable error parameters. Stimulation settings were configured at 10 Hz frequency, magnetic field intensity at 110% of the resting threshold, and a total of 1350 pulses distributed over three sequences. Every sequence incorporated 450 pulses, interspaced with 20‐s breaks, cumulating to a 285‐s duration.

To ensure consistency and minimize potential biases in the gaming performance measurements, all participants used the same standardized gaming setup during the testing. The gaming tests were conducted on computers provided by the university's computer lab, where each computer was uniformly configured. All machines were equipped with identical specifications, including the same monitor, keyboard, and mouse, ensuring that hardware‐related variables did not influence the results. Additionally, the software required for the game was preinstalled on each machine to guarantee uniformity across all test sessions. We did not allow participants to use their personal peripherals or configurations during the gaming tasks, as we aimed to control for any individual preferences or settings that might affect performance.

### 2.3 Evaluation Metrics

#### 2.3.1 Evaluation Schedule

Participants were subjected to evaluations at three pivotal time points: baseline, Day 7 (mid‐intervention), and Day 14 (endline).

### 2.4 Assessment Instruments

**FIGURE 2:**
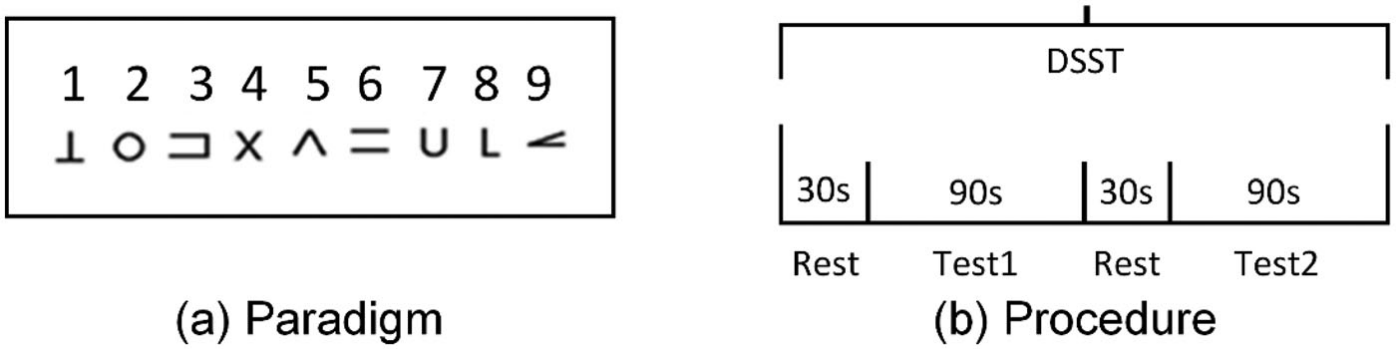
DSST Test paradigm and procedure: (a) illustration of the DSST test paradigm and (b) schematic representation of the DSST process. DSST, Digit Symbol Substitution Test.

RT and accuracy percentage (AP) of Digit Symbol Substitution Test (DSST): In conformity with the methodologies propounded by Adrian Curtin and Hasan Ayaz [19], the DSST employed computer‐mediated tests, administered in optimally illuminated experimental chambers with standardized proximities of roughly 60 cm from display units. See Figure 2 for our DSST digital methodology. The test entailed presenting participants with a repertoire of nine symbol‐number pairings. Thereafter, symbols appeared at random, necessitating the input of their numerical counterparts. Latency (with a 0.001‐s precision) and accuracy metrics were harvested post successful decoding. Emphasis was placed on response speed and overall correctness within a 90‐s timeframe. The testing protocol consisted of a rest period (30 s) followed by the DSST (90 s), and this sequence was repeated once. Outcomes were collated as mean RTs and precision percentages.

**FIGURE 3:**
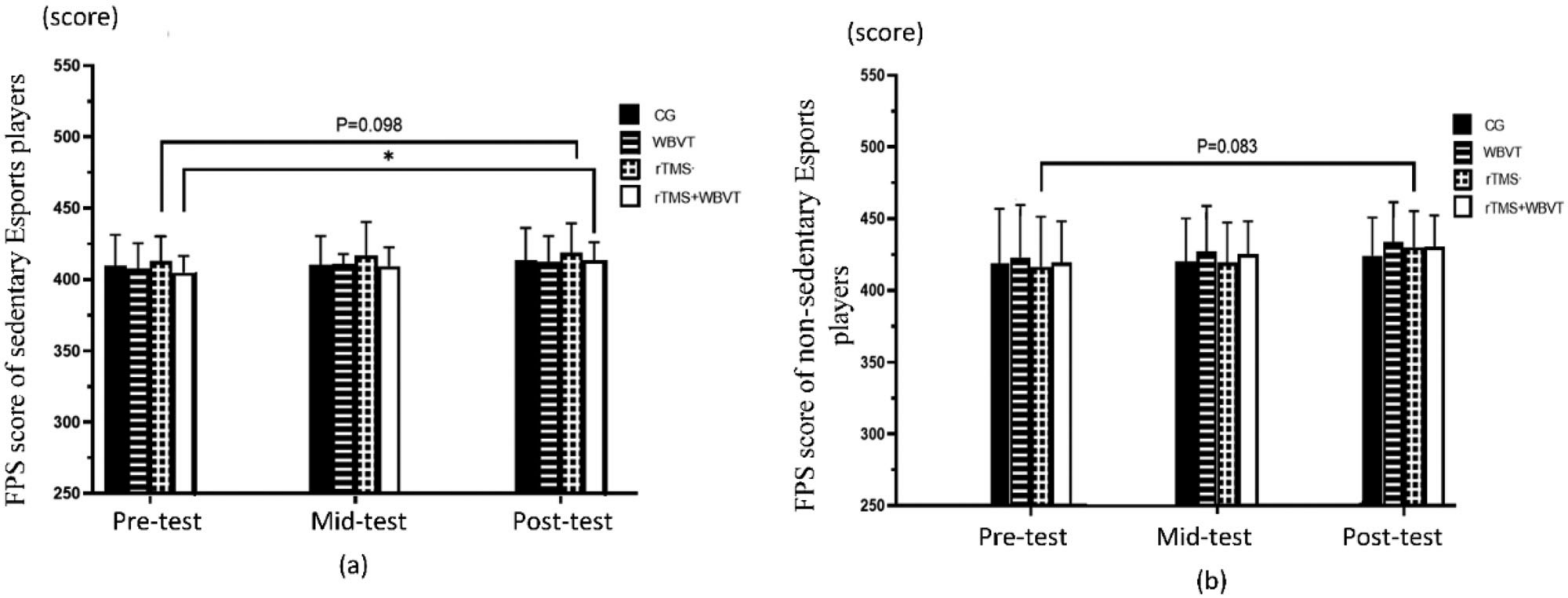
First‐person shooter (FPS) scores at the beginning, 1 week, and 2 weeks of interventions in sedentary and non‐sedentary esports playe (a) FPS scores at the beginning, 1 week, and 2 weeks of intervention in sedentary esports players. (b) FPS scores at the beginning, 1 week, and 2 weeks of intervention in non‐sedentary esports players. *P < 0.05 vs pre‐test.

First‐person shooter (FPS) performance: Utilizing the esports game “Counter‐Strike: Global Offensive,” participants engaged in single‐player matches against standardized computer artificial intelligence, a detailed visualization of which can be found in Figure 3. Players were immersed in 10‐min matches where cognitive agility, spatial awareness, and focused attention were paramount. Performance was gauged via cumulative scores attained through adversary neutralization and achievement of sub‐objectives.

### 2.5 Statistical Analysis

Data were processed using IBM SPSS Statistics Version 18.0 for Windows (IBM Corp., Armonk, NY, USA). The Kolmogorov–Smirnov test served to evaluate the normality of continuous random variable distributions. For variables exhibiting a non‐normal distribution, transformations were performed prior to hypothesis testing. Initial group comparisons of baseline variables were conducted using an independent samples *t*‐test. Subsequently, a two‐way repeated measures analysis of variance was performed to discern the differences in each dependent variable across the groups before and after the intervention training regime. This was followed by a Bonferroni post hoc comparison analysis. An independent *t*‐test was employed to probe for differences at baseline. A threshold of *p* < 0.05 was set for statistical significance.

## 3 Results

### 3.1 Baseline Measurement

From the data presented in Table 1, no significant difference in RT and AP of the DSST and FPS performance among the control, WBVT, HF‐rTMS, and HF‐rTMS + WBVT groups was seen in either sedentary esports players or non‐sedentary esports players at the baseline of the study. However, compared to RT of DSST for non‐sedentary esports players in the control group, there was an increased RT of DSST for sedentary esports players in the control group (*p* < 0.01). Similarly, a significant difference in RT was also observed between non‐sedentary esports players and sedentary esports players among the WBVT, rTMS, and rTMS + WBVT groups (all *p* < 0.01).

**TABLE 1:** Baseline variables for sedentary and non‐sedentary esports players.

Variable	Control	WBVT	HF‐rTMS	HF‐rTMS + WBVT
RT of DSST (ms)				
Non‐sedentary esports players	1555 ± 214	1547 ± 165	1560 ± 187	1559 ± 84
Sedentary esports players	1715 ± 211**	1722 ± 182**	1730 ± 130**	1712 ± 253**
AP of DSST (%)				
Non‐sedentary esports players	92.38 ± 6.04	91.31 ± 5.40	90.44 ± 6.49	89.81 ± 5.67
Sedentary esports players	92.13 ± 4.91	89.25 ± 5.50	90.94 ± 2.38	90.69 ± 3.77
FPS performance (score)				
Non‐sedentary esports players	418.56 ± 38.41	422.75 ± 39.73	416.37 ± 35.08	419.31 ± 28.86
Sedentary esports players	409.50 ± 21.79	407.63 ± 17.83	412.88 ± 20.77	404.62 ± 11.83

*Note*: The data are presented as mean ± standard deviation. **p < 0.01 versus non‐sedentary esports players.

Abbreviations: AP, accuracy percentage; DSST, Digit Symbol Substitution Test; FPS, first‐person shooter; HF‐rTMS, high‐frequency repetitive transcranial magnetic stimulation; RT, reaction time; WBVT, whole‐body vibration training

The results from the MANOVA, as shown in Table 2, reveal that the sedentary lifestyle variable did not exhibit significant differences in most multivariate tests. However, Roy's Largest Root indicated a significance level of 0.039, suggesting some influence within the sedentary group under this specific test. Regarding the intervention variable, no significant differences were detected across the multivariate tests, indicating uniformity among intervention groups at baseline. Additionally, the interaction between sedentary lifestyle and intervention displayed no significant multivariate effects, confirming that baseline characteristics were consistent across different lifestyle and intervention groups.

**TABLE 2:** Assessing baseline differences in sedentary and active lifestyle groups via MANOVA.

		*Value*	*F*	*Hypothesisd f*	*Errord f*	*Sig*.	$\eta_{p}^{2}$
Sedentary lifestyle	Pillai's Trace	0.248	1.358	10.000	96.000	0.212	0.124
	Wilks’ Lambda	0.760	1.382	10.000	94.000	0.201	0.128
	Hotelling's Trace	0.305	1.405	10.000	92.000	0.191	0.132
	Roy's Largest Root	0.267	2.559	5.000	48.000	0.039	0.210
Intervention	Pillai's Trace	0.097	0.818	6.000	96.000	0.558	0.049
	Wilks’ Lambda	0.904	0.806	6.000	94.000	0.567	0.049
	Hotelling's Trace	0.104	0.794	6.000	92.000	0.577	0.049
	Roy's Largest Root	0.078	1.255	3.000	48.000	0.300	0.073
Sedentary lifestyle × intervention	Pillai's Trace	0.485	1.024	30.000	96.000	0.448	0.242
	Wilks’ Lambda	0.565	1.036	30.000	94.000	0.433	0.248
	Hotelling's Trace	0.683	1.047	30.000	92.000	0.419	0.254
	Roy's Largest Root	0.511	1.634	15.000	48.000	0.100	0.338

### 3.2 RT and AP of DSST Across the Interventions

As shown in Table 3, RT significantly decreased from pretest to posttest in all intervention groups in sedentary esports players, with the most substantial decrease observed in the HF‐rTMS + WBVT group (from 1712 ± 253 to 1497 ± 160 ms, *p* < 0.01). Moreover, compared to the control group, only the HF‐rTMS + WBVT group showed a significantly decreased RT in sedentary esports players after a 2‐week intervention (*p* < 0.01). Compared to the baseline, non‐sedentary players also experienced reductions in RT in all intervention groups, but only the rTMS + WBVT group showed a significant decrease in RT after 2‐week intervention (from 1559 ± 84 to 1457 ± 131 ms, *p* < 0.05). AP significantly increased from pretest to posttest in all intervention groups in sedentary esports players. However, compared to the baseline, the HF‐rTMS group and the HF‐rTMS + WBVT group rather than the WBVT group displayed significant enhancement in AP after 2‐week intervention in non‐sedentary esports players. These results suggested that all these interventions, particularly the combined rTMS and WBVT intervention, effectively enhance cognitive processing speed and accuracy in esports players, with a more pronounced effect in sedentary individuals.

**TABLE 3:** The results for reaction time (RT) and accuracy percentage (AP) of Digit Symbol Substitution Test (DSST) at the beginning, 1 week, and 2 weeks of interventions in sedentary and non‐sedentary esports players.

		Sedentary esports players			Non‐sedentary esports players		
		Pretest	Mid‐test	Posttest	Pretest	Mid‐test	Posttest
RT (ms)	Control	1715 ± 211	1699 ± 225	1679 ± 197	1555 ± 214	1538 ± 186	1531 ± 168
	WBVT	1722 ± 182	1676 ± 185	1626 ± 148*	1547 ± 165	1514 ± 128	1472 ± 156
	HF‐rTMS	1730 ± 130	1664 ± 58	1594 ± 69**	1560 ± 187	1496 ± 154	1475 ± 124
	HF‐rTMS + WBVT	1712 ± 253	1638 ± 193	1497 ± 160**	1559 ± 84	1487 ± 106	1457 ± 131*
AP (%)	Control	92.13 ± 4.91	93.50 ± 3.27	92.81 ± 5.14	92.38 ± 6.04	94.19 ± 1.91	94.38 ± 2.39
	WBVT	89.25 ± 5.50	90.56 ± 3.20	91.44 ± 2.39*	91.31 ± 5.40	93.13 ± 3.42	93.25 ± 2.46
	HF‐rTMS	90.94 ± 2.38	91.69 ± 1.54	92.94 ± 2.57*	90.44 ± 6.49	92.31 ± 5.39	93.50 ± 3.86*
	HF‐rTMS + WBVT	90.69 ± 3.77	92.56 ± 4.16	93.00 ± 2.76*	89.81 ± 5.67	92.00 ± 4.53	93.19 ± 3.99*

*Note*: The data are presented as mean ± standard deviation. *p < 0.05, **p < 0.01 versus non‐sedentary esports players.

Abbreviations: HF‐rTMS, high‐frequency repetitive transcranial magnetic stimulation; WBVT, whole‐body vibration training.

To assess the potential interactive effects on participant outcomes, we conducted a principal component analysis to examine the interactions between time points, sedentary lifestyle, and various interventions (shown in Table 4). The interaction between time points and sedentary lifestyle was found to have a minimal and nonsignificant effect (*F* = 1.172, *p* = 0.063, *η* ^2^ = 0.008), suggesting that the effect of time on cognitive outcomes was largely similar for both sedentary and non‐sedentary participants. However, a more pronounced interaction was observed between time points and the types of interventions (*F* = 4.618, *p* = 0.0467, *η* ^2^ = 0.015), indicating that the interventions had a significant impact on participants’ outcomes over time. This effect suggests that different interventions produced varying outcomes across the time points, with certain interventions showing more substantial improvements than others. The interaction between sedentary lifestyle and interventions also demonstrated a significant effect (*F* = 3.529, *p* = 0.0318, *η* ^2^ = 0.017), revealing that the impact of the interventions was influenced by participants' sedentary or non‐sedentary lifestyles. Specifically, sedentary esports players appeared to benefit more from the interventions compared to their non‐sedentary counterparts. The most substantial interaction occurred between time points, sedentary lifestyle, and interventions (*F* = 7.325, *p* = 0.0483, *η* ^2^ = 0.013), indicating significant combined effects of these factors on the measured outcomes. This suggests that the combined effects of time, lifestyle, and intervention were crucial in shaping cognitive and gaming performance, with the greatest improvements observed in sedentary players undergoing interventions.

**TABLE 4:** Principal component analysis of time, lifestyle, and intervention effects.

	*F*	*Sig*.	$\eta_{p}^{2}$
Time point × sedentary lifestyle	1.172	0.063	0.008
Time point × intervention	4.618	0.0467	0.015
Sedentary lifestyle × intervention	3.529	0.0318	0.017
Time point × sedentary lifestyle × intervention	7.325	0.0483	0.013

### 3.3 FPS Scores Across Interventions

As shown in Table 5 and Figure 3, although there were numerical improvements in FPS scores from pretest to posttest across all intervention groups in sedentary esports players, only the rTMS + WBVT group showed a significant increase (*p* < 0.05). Compared to the baseline, non‐sedentary players also displayed enhancements in the FPS scores in all intervention groups; however, no significant difference was seen in all intervention groups. The analysis found no significant interactions between time points and sedentary lifestyle, time points and intervention, or the combined effects of time point, sedentary lifestyle, and intervention, with *F* values of 1.471, 0.412, and 1.325, respectively, and corresponding significance levels of 0.294, 0.671, and 0.323.

**TABLE 5:** Variance analysis results of first‐person shooter (FPS) scores at the beginning, 1, and 2 weeks of interventions in sedentary and non‐sedentary esports players.

FPS (Score)	Sedentary esports players			Non‐sedentary esports players		
	Pretest	Mid‐test	Posttest	Pretest	Mid‐test	Posttest
Control WBVT HF‐rTMS HF‐rTMS + WBVT	409.50 ± 21.79 407.63 ± 17.83 412.88 ± 20.77 404.62 ± 11.83	410.31 ± 19.99 410.87 ± 16.91 416.75 ± 23.55 409.31 ± 13.29	413.50 ± 22.53 412.44 ± 17.96 418.75 ± 20.57 413.56 ± 12.49*	418.56 ± 38.41 422.75 ± 39.73 416.37 ± 35.08 419.31 ± 28.86	419.81 ± 30.63 426.94 ± 34.51 419.31 ± 27.93 425.12 ± 23.13	424.25 ± 26.56 433.44 ± 32.50 430.06 ± 25.13 430.37 ± 21.89
Main effect of *F* for the groups		0.510			0.208	
Main effect of *F* for the weeks		5.608** 0.006** 0.160			5.351* 0.019* 0.082	

*Note*: Data are presented as mean ± standard deviation. *p < 0.05, **p < 0.01 versus pretest.

Abbreviations: HF‐rTMS, high‐frequency repetitive transcranial magnetic stimulation; WBVT, whole‐body vibration training.

Table 6 provides a principal component analysis for FPS scores, showing the interactions between time points, sedentary lifestyle, and interventions. Although there were numerical improvements in FPS scores across all interventions, no significant interaction effects were observed between time points and sedentary lifestyle (*F* = 1.471, *p* = 0.294, *η* ^2^ = 0.109) or between time points and interventions (*F* = 0.412, *p* = 0.671, *η* ^2^ = 0.065). Similarly, the interaction between sedentary lifestyle and interventions was not significant (*F* = 0.765, *p* = 0.418, *η* ^2^ = 0.113). These results indicate that, although interventions led to numerical improvements in FPS scores, these changes were not statistically significant, and there were no substantial differences between sedentary and non‐sedentary players in terms of their gaming performance.

**TABLE 6:** First‐person shooter (FPS) scores principal component analysis of time, lifestyle, and intervention effects.

	*F*	*Sig*.	$\eta_{p}^{2}$
Time point × sedentary lifestyle	1.471	0.294	0.109
Time point × intervention	0.412	0.671	0.065
Sedentary lifestyle × intervention	0.765	0.418	0.113
Time point × sedentary lifestyle × intervention	1.325	0.323	0.103

## 4 Discussion

The present study explored the impacts of HF‐rTMS and WBVT on the cognitive efficiency of esports players, distinguishing between sedentary and non‐sedentary behaviors. Our results indicated that both interventions, conducted individually or combinedly, led to improvements in cognitive variables, including DSST response time, AP, and FPS scores. These enhancements were more pronounced in sedentary esports players than in non‐sedentary esports players. Thus, WBVT and HF‐rTMS may offer notable cognitive benefits, especially for players with sedentary lifestyles, potentially offsetting the cognitive downsides of sedentariness.

To the current knowledge, the present study was the first to study the cognitive impact of sedentary behavior in esports athletes and identified a significant reduction in cognitive performance among sedentary participants compared to their active peers. Our finding highlighted the detrimental effects of prolonged sitting on cognitive functions, especially in environments demanding high cognitive engagement, such as competitive gaming. This research, pioneering in its focus on esports athletes, added insights to the understanding of cognitive health in the context of sedentary lifestyles, contributing to both cognitive science and esports disciplines.

Our study found significant improvements in RT and AP of the DSST across various interventions and time points. Among sedentary esports players, those undergoing WBVT exhibited a notable reduction in RT, from 1722 ± 182 ms at baseline to 1626 ± 148 ms at posttest (*p* < 0.01), suggesting that this intervention enhances cognitive processing speed. Similarly, in the HF‐rTMS group, RT decreased from 1730 ± 130 ms at baseline to 1594 ± 69 ms (*p* < 0.01) at posttest, indicating that NIBS techniques also have a positive impact on cognitive performance. The combined WBVT and HF‐rTMS intervention resulted in the most significant reduction in RT, from 1712 ± 253 ms at baseline to 1497 ± 160 ms (*p* < 0.01), emphasizing the potential synergistic effects of combining these interventions. Non‐sedentary esports players also showed improvements in RT with WBVT or HF‐rTMS, though statistical significance was only observed in the combined group, where RT decreased from 1559 ± 84 ms at baseline to 1457 ± 131 ms (*p* < 0.05) at posttest. These findings suggest that these interventions may have a greater impact on individuals with lower baseline cognitive performance. AP scores showed consistent increases for both sedentary and non‐sedentary players, with the most substantial improvement in sedentary players undergoing HF‐rTMS + WBVT, from 90.69% ± 3.77% at baseline to 93.00% ± 2.76% at posttest (*p* < 0.05), and in non‐sedentary players, from 89.81% ± 5.67% at baseline to 93.19% ± 3.99% (*p* < 0.05). These improvements highlight the potential of these interventions to enhance attention and information‐processing skills, which are essential for gaming performance.

The combined use of HF‐rTMS and WBVT offers significant practical implications for esports training, especially in improving cognitive functions, such as processing speed, attention, and decision‐making. Esports organizations could incorporate these interventions into training regimens to enhance cognitive performance, particularly for sedentary players. The synergistic effects observed may stem from the complementary actions of HF‐rTMS, which targets brain regions linked to cognitive processing, and WBVT, which may enhance neuroplasticity. However, future studies should address potential confounding variables like sleep and nutrition and explore the applicability of these interventions in other populations.

In terms of FPS scores, the study revealed a general trend of improvement over time for both sedentary and non‐sedentary esports players. Specifically, participants in the WBVT, HF‐rTMS, and combined intervention groups exhibited substantial gains in FPS scores. Sedentary esports players in the combined HF‐rTMS and WBVT group demonstrated the most significant improvement in FPS scores, suggesting that a combined intervention of WBVT and HF‐rTMS may have more profound impact on gaming performance. Non‐sedentary esports players also benefited from these interventions, although no significance in FPS scores was observed after 2 weeks of interventions.

A significant highlight and novel contribution of this study is the exploration of the combined effects of WBVT and HF‐rTMS. Previous studies had reported that each of WBVT and HF‐rTMS intervention could enhance cognitive function. For instance, Goudarzian et al. (2017) demonstrated the efficacy of WBVT in improving cognitive function among the elderly, whereas highlighted the cognitive enhancement benefits of HF‐rTMS in individuals with depression (Kim et al. 2019). However, the exploration of their combined effects, especially in the context of esports players, was lacked. Consistent with the previous studies, the present study showed that both interventions individually enhanced cognitive outcomes in esports players; in particular, the combined intervention exhibited a more pronounced benefit, especially in FPS metrics. This finding suggested a possible synergistic effect when both modalities are simultaneously administered, which could have profound implications for designing cognitive enhancement strategies for esports players and potentially other populations as well.

Enhanced cognitive function and FPS metrics intimate the potential utility of tailored modalities, such as WBVT and HF‐rTMS in elite esports training. Organizations and mentors in esports are advised to assimilate these techniques to augment reaction speeds, concentration, and comprehensive gaming prowess. Of particular note is the efficacy of these interventions for sedentary esports participants striving to equate their active peers. Fundamentally, this research augments the expanding scientific compendium geared toward refining esports competitiveness. As esports metamorphoses rapidly, leveraging evidence‐led strategies such as WBVT and HF‐rTMS will be instrumental for participants and entities aiming to sharpen their competitive prowess and spearhead esports ingenuity. This research accentuates the essence of empirically grounded training in sustaining an advantage in this evolving, skill‐intensive domain.

A key consideration for future research is how these interventions could be integrated into existing esports training regimens. Although the current study focused on cognitive enhancements, future work should explore how HF‐rTMS and WBVT could be incorporated into regular training schedules for esports players, potentially improving sustained cognitive performance during intense practice or tournament settings. Additionally, the impact of these interventions on actual competition outcomes and tournament performance remains to be explored. It is also important to acknowledge that different esports genres require distinct cognitive skills, which may influence the effectiveness of these interventions.

## 5 Conclusions

In the ever‐evolving domain of cognitive enhancement research, our study stood out by introducing a novel, combined intervention approach tailored for esports players. Our findings illuminated the profound synergistic effects of WBVT and HF‐rTMS in enhancing cognitive outcomes. The study not only solidified the individual merits of WBVT and HF‐rTMS but also accentuated their compounded benefits, particularly evident in FPS metrics. By focusing on esports players, a demographic with unique cognitive demands, we had underscored the adaptability and potential of these interventions across diverse populations. As esports continues its meteoric rise in global popularity and significance, our research offers a foundational approach to optimizing player performance through targeted cognitive interventions. Beyond its direct applications, this research serves as a beacon for future endeavors, emphasizing the importance of tailoring interventions to specific population needs and exploring the untapped potential of combined therapeutic modalities.

## 6 Study Limitations

Although our study offers valuable insights into cognitive enhancement strategies for esports players, several limitations should be considered. One key limitation is the focus on esports players as the study cohort, which, while providing detailed insights into this specific group, may limit the generalizability of the findings to other populations. Additionally, potential confounding factors such as participants’ nutritional intake, sleep patterns, and other lifestyle elements were not rigorously controlled, which could have impacted cognitive outcomes.

Furthermore, we did not account for time‐of‐day effects on cognitive performance, which could introduce variability due to natural fluctuations in cognitive abilities throughout the day. Although participants met the minimum requirement of 5 h of weekly gaming, we did not consider the overall years of experience or the intensity of their gaming practice, both of which may have influenced baseline cognitive performance. Additionally, the study did not control for the specific game genres in which players specialized, potentially contributing to performance differences due to the distinct cognitive demands of various game types. Future research should address these factors to offer more precise and comprehensive assessments. Future studies could also consider incorporating a wider range of games, including the recently released CS:GO and CS2 (Sharpe et al. 2023, 2024), to better reflect the current gaming landscape. Furthermore, future research should include more detailed protocol standardization for FPS testing and gather qualitative player feedback. A limitation of the current study is the absence of effect size calculations for all significant findings and the lack of analysis on potential confounding variables, which should be addressed in future investigations. Another limitation is the uneven gender distribution among participants, with fewer female participants, which prevented a detailed analysis of gender differences in response to the interventions. Future research should aim to achieve a more balanced gender representation in order to explore potential gender‐based differences in the effectiveness of these interventions.

## Author Contributions

**Shan He**: methodology, validation, writing–review and editing, writing–original draft. **Lu Leng**: writing–original draft, writing–review and editing, methodology. **Dongdong Gao**: formal analysis, validation, methodology, data curation. **Yu Chen**: methodology, validation, visualization, writing–original draft. **Weiji Deng**: validation, methodology. **Jiarui Wu**: methodology, validation. **Peilun Li**: methodology, validation, software. **Yilin Chen**: methodology, validation, software. **Jinglin Huang**: validation, methodology, writing–original draft. **Guoqing Liu**: visualization, validation, writing–original draft. **Jiarui Su**: validation, visualization, formal analysis. **Jianwei Peng**: validation, visualization, software. **Wenhuang Guo**: software, formal analysis, validation. **Junfeng Zhang**: software, formal analysis, data curation. **Junhao Huang**: software, validation, methodology, writing–review and editing, writing – original draft, funding acquisition, project administration.

## Ethics Statement

The present study was approved by the Ethics Committee of Guangzhou Sport University (2020LCLL‐008), registered in the Chinese Clinical Trial Registry (10/09/2021), and the registration number is ChiCTR2100051029 and was conducted according to the declaration of Helsinki.

## Consent

The fully informed participants consented to all the procedures that were performed as well as with the publication of the details written in this article. They were informed about the possibility to withdraw from participation at any time without consequences.

## Conflicts of Interest

The authors declare no conflicts of interest.

### Peer Review

The peer review history for this article is available at https://publons.com/publon/10.1002/brb3.70473

## Supporting information



Supporting Information

## Data Availability

The datasets generated during the current study are not publicly available as this conflict with our ethics agreement with the participants. Requests to obtain confidential access to the datasets should be directed to the corresponding author.
