# Supplementary material for: Combined Effect of HF‐rTMS and Whole‐Body Vibration Exercise on Cognitive Efficiency in Esports Players With or Without Sedentary Behaviors: A Randomized Controlled Trial
Source: Brain Behav. 2025 May 8;15(5):e70473. doi: 10.1002/brb3.70473 (PMC12060219; doi:10.1002/brb3.70473)
Supplement: Supplementary file 1 — Supporting Information [file BRB3-15-e70473-s001.pdf]

Supplementary Material

This physical activity scale is a comprehensive questionnaire designed to assess an individual's walking habits, participation in moderate and vigorous physical activities, and sedentary behaviors over the past week, including both workdays and rest days.

1. In the past 7 days, how many days did you go for a walk for more than 10 minutes?
   ( ) days/week ☐ Did not go for a walk → Skip to question 2
2. How much time do you spend walking each day?
   ( ) hours/day ( ) minutes/day
3. How long have you maintained the above "1,2" status?
   1-3 months ( ) 3-6 months ( ) 6 months-1 year ( ) Over 1 year ( )
4. In the past 7 days, how many days did you participate in vigorous-intensity physical activities (such as aerobic fitness, running, fast cycling, swimming, football, basketball, etc.), lasting more than 10 minutes? (Exclude the walking time you already described)
   ( ) days/week ☐ No vigorous-intensity physical activity → Skip to question 5
5. How much time do you spend each day on vigorous-intensity physical activities?
   ( ) hours/day ( ) minutes/day
6. How long have you maintained the above "4,5" status?
   1-3 months ( ) 3-6 months ( ) 6 months-1 year ( ) Over 1 year ( )
7. In the past 7 days, how many days did you participate in moderate-intensity physical activities (such as brisk walking, dancing, bowling, table tennis, badminton, etc., with a heart rate not less than 110 bpm), lasting more than 10 minutes?
   ( ) days/week ☐ No moderate-intensity physical activity → Skip to question 8
8. How much time do you spend each day on moderate-intensity physical activities?
   ( ) hours/day ( ) minutes/day
9. How long have you maintained the above "7,8" status?
   1-3 months ( ) 3-6 months ( ) 6 months-1 year ( ) Over 1 year ( )
10. The following questions are about the time you spent sitting, reclining, or lying down in the past period, including at work and in life, such as desk work, sitting (or reclining or lying) to chat, reading, or watching TV, surfing the internet, playing computer games, etc.
    In the past 7 days, how much time did you spend each day in the above postures on workdays?
    ( ) hours/day ( ) minutes/day
11. How long have you maintained the above "10" status?
    3-6 months ( ) 6 months-1 year ( ) Over 1 year ( ) ? months ( )
12. In the past 7 days, how much time did you spend each day in the above postures on weekends or rest days?
    ( ) hours/day ( ) minutes/day
13. How long have you maintained the above "12" status?
    3-6 months ( ) 6 months-1 year ( ) Over 1 year ( ) ? months ( )
